# The Inhibitory Effects of Ca^2+^ Channel Blocker Nifedipine on Rat Kv2.1 Potassium Channels

**DOI:** 10.1371/journal.pone.0124602

**Published:** 2015-04-20

**Authors:** Xian-Tao Li, Xiao-Qing Li, Xi-Mu Hu, Xiao-Yue Qiu

**Affiliations:** 1 Department of Neuroscience, College of Life Sciences, South-Central University for Nationalities, Wuhan, China; 2 South-Central University for Nationalities, Wuhan, China; Dalhousie University, CANADA

## Abstract

It is well documented that nifedipine, a commonly used dihydropyridine Ca^2+^ channel blocker, has also significant interactions with voltage-gated K^+^ (Kv) channels. But to date, little is known whether nifedipine exerted an action on Kv2.1 channels, a member of the Shab subfamily with slow inactivation. In the present study, we explored the effects of nifedipine on rat Kv2.1 channels expressed in HEK293 cells. Data from whole-cell recording showed that nifedipine substantially reduced Kv2.1 currents with the IC_50_ value of 37.5 ± 5.7 μM and delayed the time course of activation without effects on the activation curve. Moreover, this drug also significantly shortened the duration of inactivation and deactivation of Kv2.1 currents in a voltage-dependent manner. Interestingly, the half-maximum inactivation potential (*V*_1/2_) of Kv2.1 currents was -11.4 ± 0.9 mV in control and became -38.5 ± 0.4 mV after application of 50 μM nifedipine. The large hyperpolarizing shift (27 mV) of the inactivation curve has not been reported previously and may result in more inactivation for outward delayed rectifier K^+^ currents mediated by Kv2.1 channels at repolarization phases. The Y380R mutant significantly increased the binding affinity of nifedipine to Kv2.1 channels, suggesting an interaction of nifedipine with the outer mouth region of this channel. The data present here will be helpful to understand the diverse effects exerted by nifedipine on various Kv channels.

## Introduction

Kv channels are involved in various functions of cell such as maintenance of membrane potential, repolarization of the action potential, regulation of neuronal firing patterns, synaptic integration, and neurotransmitter release [1]. Kv channels share a common structure comprising four α-subunits, and each subunit was proposed to contain six transmembrane helices (S1–S6), arranged around a central K^+^-selective pore [2, 3]. It is found that the major element of the voltage sensor is located at S4 region of these channels [4–6]. Movement of the S4 region together with secondary movements of the S2 and S3 regions after depolarization initiated activation of Kv channels [7–9].

Kv2.1 channels, a member of the Shab subfamily of Kv channels, are expressed in a wide variety of excitable cells, such as skeletal muscle [10], neurons [11–13], cardiomyocytes [14, 15]. They also play crucial physiological roles in maintaining membrane potential and modulating electrical excitability in neurons and muscle [16, 17]; therefore, Kv2.1 channels are subject to intensive studies for several years. The delayed rectifier K^+^ currents which are carried by Kv2.1 channels display outward rectification with slow inactivation, at least on a second time scale, in neuronal cells [18]. Such slow inactivation is regarded as “C-type” inactivation, which is insensitive to N-terminal deletion [19].

The dihydropyridines nifedipine, a Ca^2+^ channel antagonist, is widely used as a medicine for a variety of cardiovascular diseases, such as hypertension [20, 21] and angina pectoris [22]. Previous studies indicated that nifedipine also inhibited macroscopic currents through heterologously expressed human Kv1.5 [23] and rat Kv4.3 channels [24]. In native cellular preparations, people demonstrated that nifedipine present inhibitory effects on transient outward K^+^ current (I_to_) in rabbit atrial [25] and rat ventricular cells [26], as well as ultra-rapid delayed rectifier K^+^ current (IKur) in human atrial cells [27]. Furthermore, it was reported that other antagonists of Ca^2+^ channels such as verapamil and diltiazem exerted a blocking effect on Kv channels. Verapamil led to the high-affinity block of currents through HERG channels expressed in HEK293 cells [28]. Similarly, diltiazem showed suppressive actions on I_to_ and IKur in a concentration-dependent manner in human atrial myocytes [27]. Currently, the exact mechanism underlying inhibitory effects of those chemicals on Kv channels are largely unknown.

HERG channels were identified as the molecular basis of rapid activating component (I_Kr_) of cardiac delayed rectifier K^+^ currents [29]. One of the I_to_ currents in native heart cell was encoded by rapidly activating Kv1.5 channels [30, 31]. Nifedipine-mediated reduction of both HERG and Kv1.5 channels could lead to the suppressions of both I_Kr_ and I_to_ currents in cardiomyocytes. Consequently, the prolonged action potential duration and the increased refractory period could result in development of cardiac arrhythmias [32]. Therefore, it is very important to understand the diverse effects of nifedipine on various Kv channels. It is known that several distinct Kv currents were carried by Kv2.1 channels. In particular, slowly inactivating K^+^ currents (I_K, slow_) which were contributed to repolarization process in mouse cardiomyocytes were encoded by delayed rectifier Kv2.1 channels [17, 33, 34]. In mammalian sympathetic neurons, delayed rectifier K^+^ currents which are encoded by Kv2.1 channels played distinct roles in regulating the excitability of these neurons [16]. Kv2.1 channels also regulated rat hippocampal somato-dendritic excitability during episodes of the high-frequency synaptic transmission [35]. These reports suggested that Kv2.1 channels play crucial physiological and pathophysiological roles in different tissues. Although Ca^2+^ channel antagonist nifedipine has inhibitory actions on some kinds of Kv channels, currently, there are not any reports about the effects of nifedipine on Kv2.1 channels. In the study here, patch-clamp technique was employed to explore the possible actions of nifedipine on macroscopic currents through heterologously expressed Kv2.1 channels in HEK293.

## Methods

### Cell culture and the transfection of Kv2.1 plasmids

HEK293 cells were grown in Dulbecco’s modified Eagle’s medium (DMEM), supplemented with 10% fetal calf serum, 100U/ml penicillin and 100ug/ml streptomycin at 37°C in 95% humidified air with 5% CO_2_. The medium was replaced every 2 days, and confluent cells were digested with 0.25% trypsin and split at a rate of 1:2–1:3 every 4 days. Then, cells were resuspended and moved into 24-well plates containing glass coverslips. Before transfection, cells were maintained in the incubator for growing for 24 h.

Rat Kv2.1 subcloned into pEYFP-N1 vector (Clontech**)** by ligating into the XhoI/EcoRI sites was generously provided by Dr. Len Kaczmarek (Yale University, the School of Medicine, New Haven, CT). Mutations of Kv2.1 channels were performed with the QuikChange Site-directed Mutagenesis Kit (Stratagene, LaJolla, CA) according to the manufacturer’s protocol. Sequence analysis was undertaken to confirm mutation. Plasmids were transfected into HEK293 cells using Lipofectamine 2000 (Life Technologies, Bethesda, MD) according to the manufacturer’s protocol. Briefly, Plasmids and Lipofectamine 2000 were diluted in 50μl of Opti-MEM without serum, respectively. After 5 min incubation at room temperature, the diluted plasmids were combined with diluted Lipofectamine 2000. The total 100 μl of complexes were added into 24-well plates and HEK293 cells were grown for 24–48 h in incubator before transferring into recording chamber for patch-clamp measurement.

### Electrophysiology

The effects of nifedipine on whole-cell currents from heterologously expressed Kv2.1 channels in HEK293 cells were assessed by patch-clamp recording. EPC-9 patch-clamp system (HEKA Elektronik, Lambrecht, Germany) was employed for data acquisition and analyses. Patch pipettes were pulled from thin-walled borosilicate glass capillary tubes by P-97 puller (Sutter Instrument). The bath solution contained (in mM): 75 Na-gluconate, 70 NaCl, 5 KCl, 5 HEPES, 5 glucose, with pH adjusted to 7.4 using NaOH; the pipette solution contained (in mM): 150 KCl, 5 HEPES, 5 EGTA, 5 Glucose, 5 Na_2_ATP, with pH adjusted to 7.3 using KOH. The coverslips with Kv2.1-transfected HEK293 cells were mounted on the stage of an inverted microscope (Olympus IX 71), and only cultured cells with fluorescence were chosen for patch-clamp recording. The currents were low pass filtered at 2 kHz (-3dB) and sampled at 5 kHz. A holding potential of -80 mV was standard unless otherwise noted. Capacity compensation and 90% series resistance compensation were routinely performed in all measurements. All experiments were conducted at room temperature (22°C).

### Analysis and statistics

Concentration-response curves were acquired by application of 1, 10, 50, 100 and 500 μM nifedipine. Responses were plotted relative to the control. The response function was fitted by the Hill equation: *y* = *I*_min_ + (*I*_max_−*I*_min_) / (1 + (IC_50_/*x*) ^*h*^), where *I*_max_ and *I*_min_ are current with maximum and minimum, respectively, IC_50_ is the concentration for 50% block, and *h* is the Hill coefficient.

The decay traces of the currents evoked by long (15 s) depolarizing voltage steps to test potentials from a holding potential of -80 mV were fitted by the single exponential using the following expression: *f*(*t*) = *A* exp (−*t* /*τ*) + *A*_ss_, where *t* is time, *τ* is the time constants of decay of the inactivating Kv2.1 currents, *A* is the amplitudes of the inactivating current components, and *A*_ss_ is the amplitude of the steady state, noninactivating component of the outward current. Time zero was set at the peak of the outward current for all fits.

To build the activation and steady-state inactivation curve, related data were obtained by performing stimulation protocols described in Results section. These data were fitted by a Boltzmann equation: *I* ⁄ *I*_max_ = 1 ⁄ [1 + exp (*V*_1⁄2_−*V*) ⁄ *k*], where *V*_1⁄2_ is the half-maximal activation potential for activation gate or the half-maximal inactivation potential for inactivation gate, *V* is the conditioning potential, and *k* is the slope factor.

Unless otherwise noted, results are expressed as mean ± SE. Statistical significance was calculated using paired Student’s t-test or one-way analysis of variance (ANOVA). A value of P < 0.05 was accepted for the statistical difference.

## Results

### Nifedipine reversibly inhibited Kv2.1 currents

**Fig 1:**
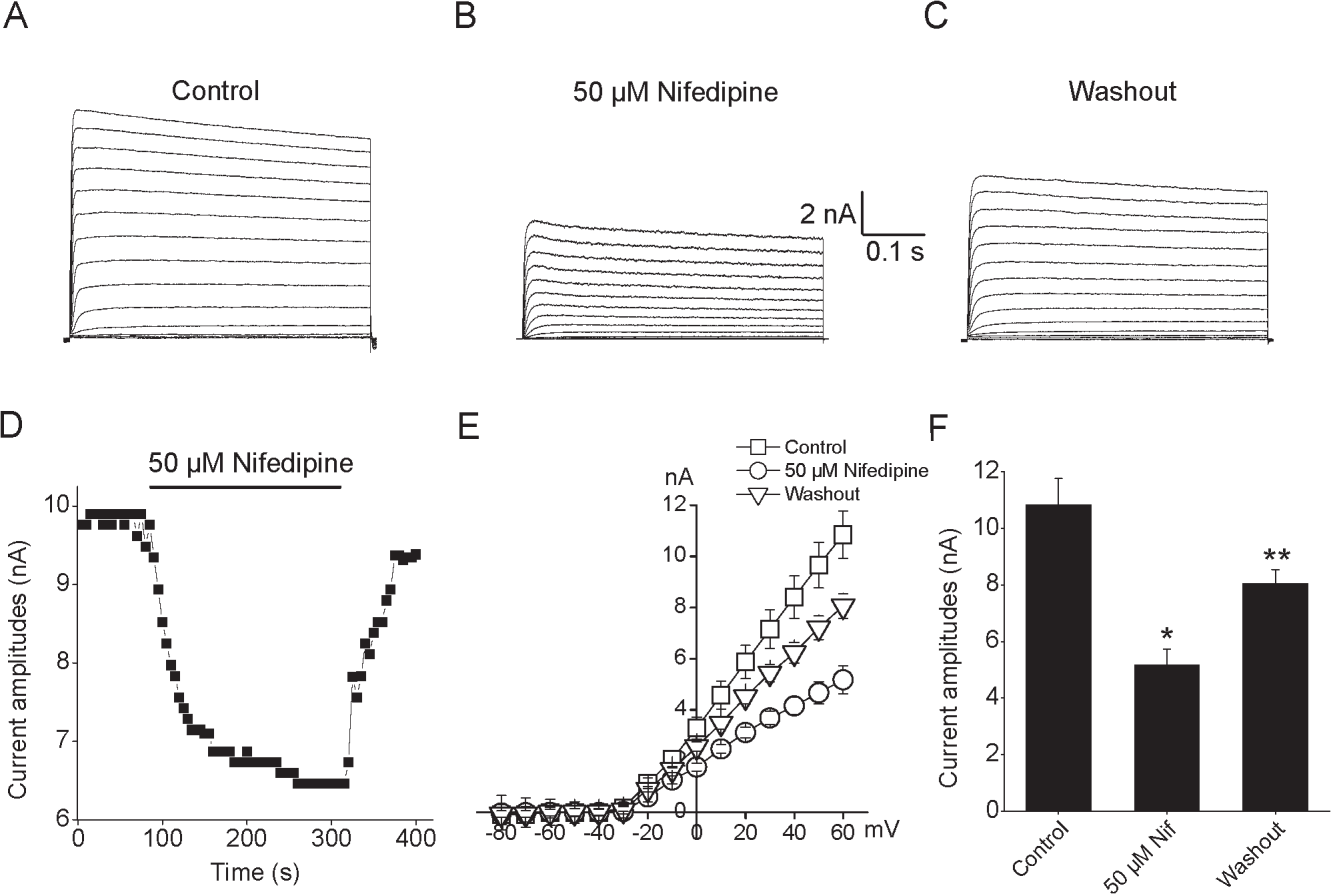
Nifedipine reversibly inhibited macroscopic Kv2.1 currents. Whole-cell Kv2.1 currents were elicited during 500 ms voltage steps to potentials ranging from -80 to +60 mV in the absence of nifedipine. (B) Exposure to 50 μM nifedipine significantly reduced the Kv2.1 currents. (C) After washout, Kv2.1 currents were partial and significant recovery. (D) The time course of inhibitory actions of nifedipine (n = 6). (E) Current-voltage (I-V) curves of the absence, presence and washout of 50 μM nifedipine (n = 11). (F) Statistical analysis of nifedipine effects at +60 mV (n = 11). *P <0.05 compared with control group and ** P <0.05 compared with the group of nifedipine treatment.

To examine the effects of nifedipine on Kv2.1 currents, rat Kv2.1 channels were heterologously expressed in cultured HEK293 cells. Macroscopic Kv2.1 currents were routinely elicited by a 500 ms voltage steps to potentials from -80 mV to +60 mV at a holding potential of -80 mV (Fig 1A). Extracellular application of 50 μM nifedipine potentially inhibited the Kv2.1 currents by 52.8% at +60 mV (Fig 1B and 1E; n = 11, P<0.05). After wash out to control solution, Kv2.1 currents were restored to 75.3% of control current levels (n = 9, P<0.05), suggesting that the effects of nifedipine on these currents were partially reversible (Fig 1C and 1E). To obtain the time course of Kv2.1 current suppression by 50 μM nifedipine, a 50 ms voltage step to +40 mV from a holding potential of -80 mV was repeatedly used to evoke macroscopic currents. A typical time course of onset as well as of the reversal of effect was shown in Fig 1D.

**Fig 2:**
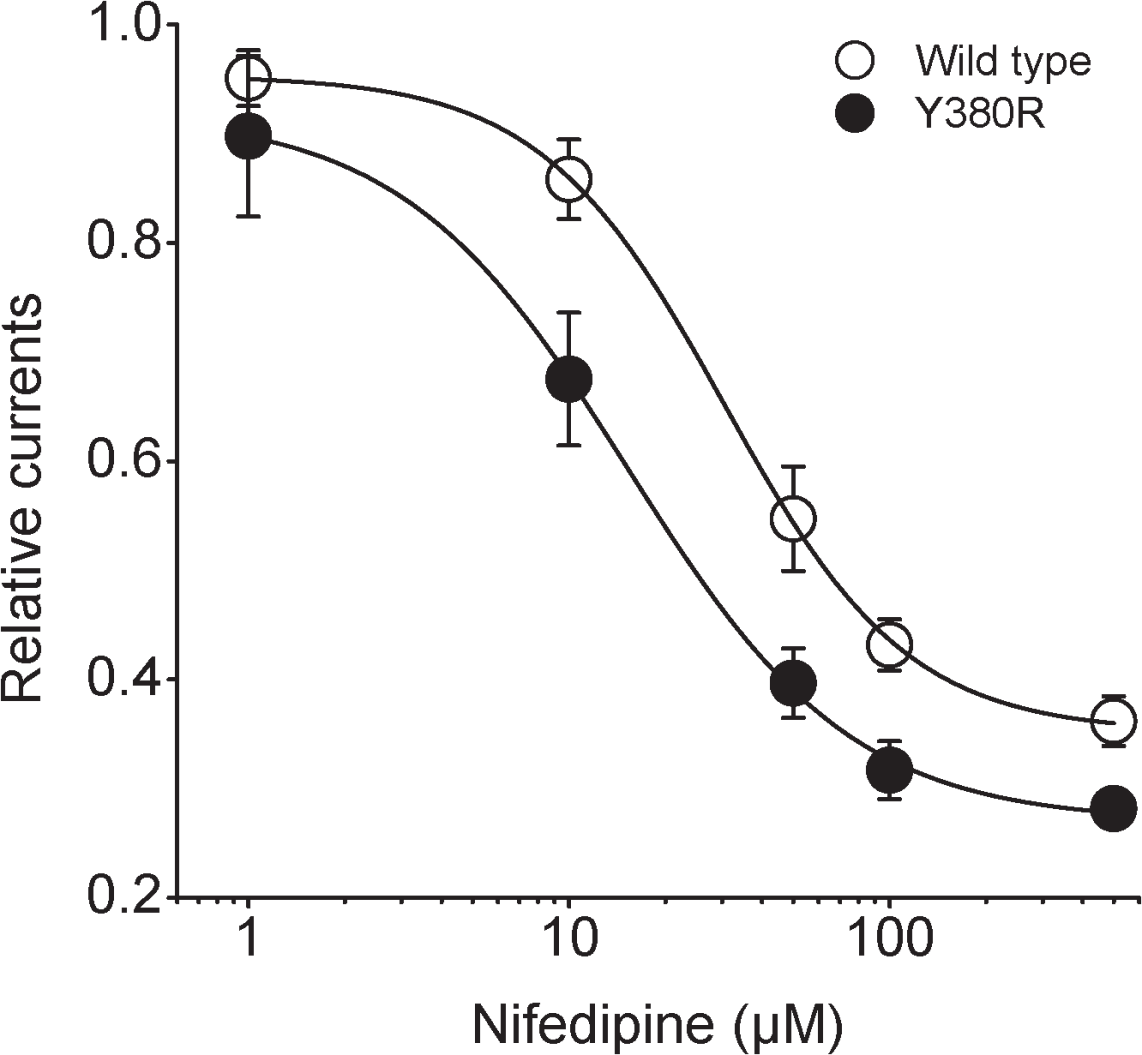
Concentration–response curves for the effects of nifedipine on wild type and Y380R mutant of Kv2.1 channels. The protocol of stimulation is identical to that in Fig 1. Whole-cell currents at +60 mV were plotted against the concentration of nifedipine. Solid lines were fit to the data of wild type Kv2.1 currents using a Hill equation with IC_50_ value of 37.5 μM and *h* value of 0.9 (n = 8). For Y380R-Kv2.1 currents, the value of IC_50_ is 15.2 μM and value of *h* is 1.1 (n = 7).

In addition, nifedipine suppressed Kv2.1 currents in a concentration-dependent way. After exposure to different doses of nifedipine, data were collected and normalized to control currents for building the concentration-response curves (Fig 2). The normalized data were well fitted by Hill equation with the IC_50_ value of 37.5 ± 5.7 μM (n = 8). Previous report revealed that the substitution of valine or tyrosine for arginine at Kv1.5 position 487 can lower the binding affinity of nifedipine to this channel [36], suggesting that the interactions with the outer mouth region of this channel underlie the effects of nifedipine. However, the residue of the shab channel Kv2.1 at the position equivalent to Kv1.5 487 is tyrosine. Thus, we speculated that substitution of tyrosine with arginine at Kv2.1 position 380 could result in an alteration in the channel affinity of nifedipine. Mutagenesis in Kv2.1 channels was made and subsequently the concentration-response curves for the action of nifedipine on Y380R-Kv2.1 currents were plotted in Fig 2 (n = 7). The corresponding IC_50_ value was reduced to 15.2 ± 1.3 μM, suggesting that the effects of nifedipine on this mutant were significantly decreased compared with wild type Kv2.1 channels. This evidence also implied that nifedipine can exert its action by interacting with the outer mouth region of Kv2.1 channels.

### Effects of nifedipine on the activation of Kv2.1 currents

**Fig 3:**
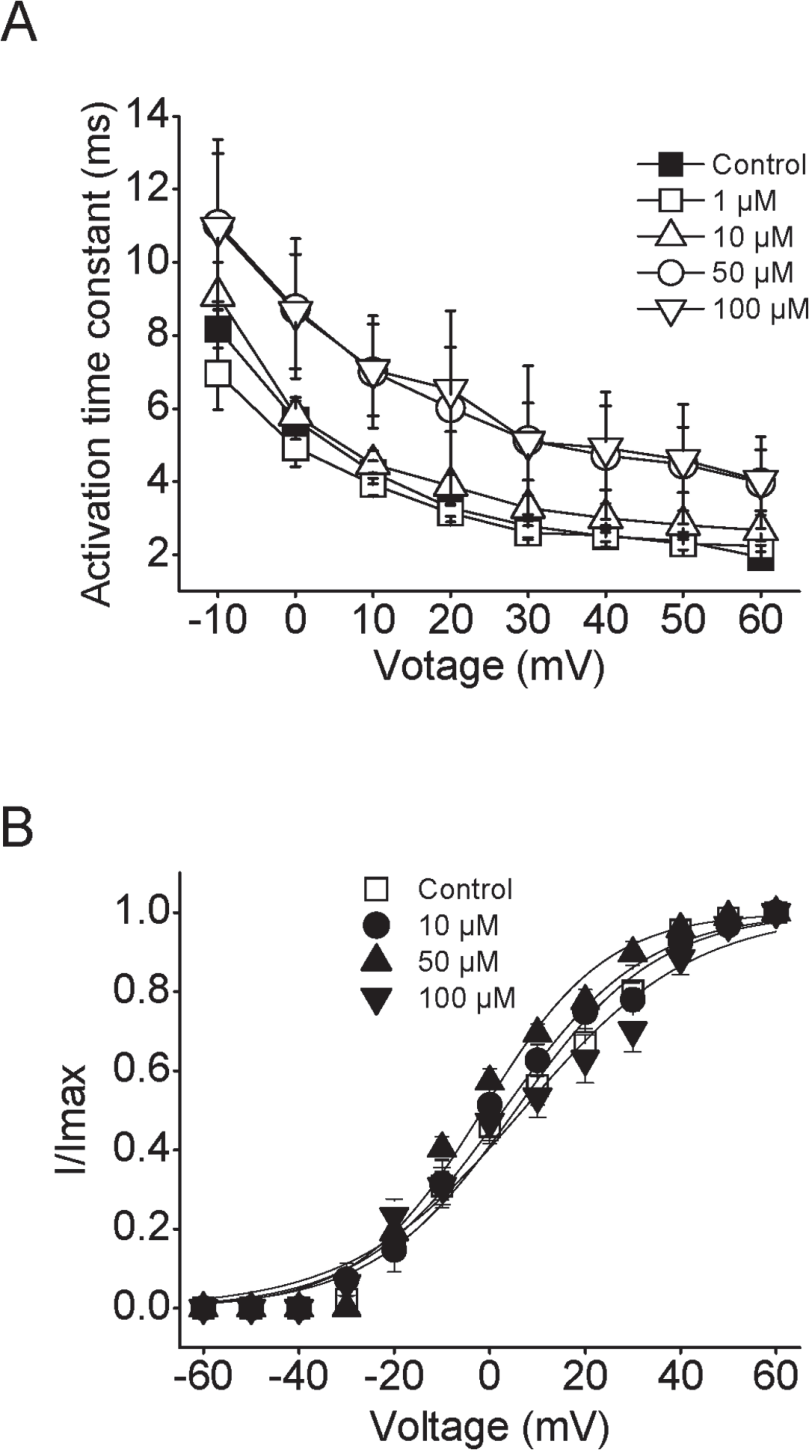
Effects of nifedipine on the activation of Kv2.1 currents. (A) The voltage dependence of activation time constant was altered by the different concentrations of nifedipine (n = 9). (B) Activation curves of Kv2.1 currents during each test pulse in the absence and presence of different levels of nifedipine were normalized to maximal currents (*I*/*I*_max_) and plotted against conditioning prepulse potential. Data are fitted with a Boltzmann equation (n = 7).

As described previously [37], the shape of activation time course of Kv2.1 channels was a typical sigmoid. Activation time constants (τ_act_) were obtained by fitting the raising phase of these current traces which were evoked by voltage steps to potentials from -10 mV to +60 mV at a holding potential of -80 mV. As marked in Fig 3A, the time course of Kv2.1 current activation was voltage dependent as noted previously [38]. For example, the value of τ_act_ in control is 8.2 ± 0.5 ms at -10 mV, and it became 2.5 ± 0.3 ms at +40 mV (n = 9, P<0.05), suggesting that depolarization accelerated the Kv2.1 current activation. Moreover, nifedipine increased the τ_act_ in a dose-dependent manner. In the presence of 50 and 100 μM nifedipine, the values of τ_act_ were significantly increased to 4.7 ± 1.3 ms and 4.9 ± 0.1 ms at +40 mV (n = 9, P<0.05), respectively. But no significant effect was found after application of 1 and 10 μM nifedipine (n = 9, P>0.05). Certainly, these data suggested that high dose of nifedipine delayed the activation time course of Kv2.1 currents. Previous works revealed that the activation of Kv currents was prolonged by the closed-channel blocker [39]. In agreement with this notion, nifedipine was expected to bind the Kv2.1 in its closed state.

To plot the activation curves, a dual-pulse protocol was used in which a test potential of -40 mV was preceded by a conditioning prepulse of different potentials. Collected raw data were normalized and then fitted by a single Boltzmann equation (Fig 3B). The value of *V*_1/2_ is 5.7 ± 2.3 mV at control, 6.8 ± 1.9 mV at 10 μM, 3.8 ± 1.9 mV at 50 μM and 9.5 ± 5.3 mV at 100 μM nifedipine (n = 7). No statistical difference between these values of *V*_1/2_ indicated that nifedipine did not change the activation gating of Kv2.1 currents (n = 7, P>0.05).

### Voltage-dependent block by nifedipine

**Fig 4:**
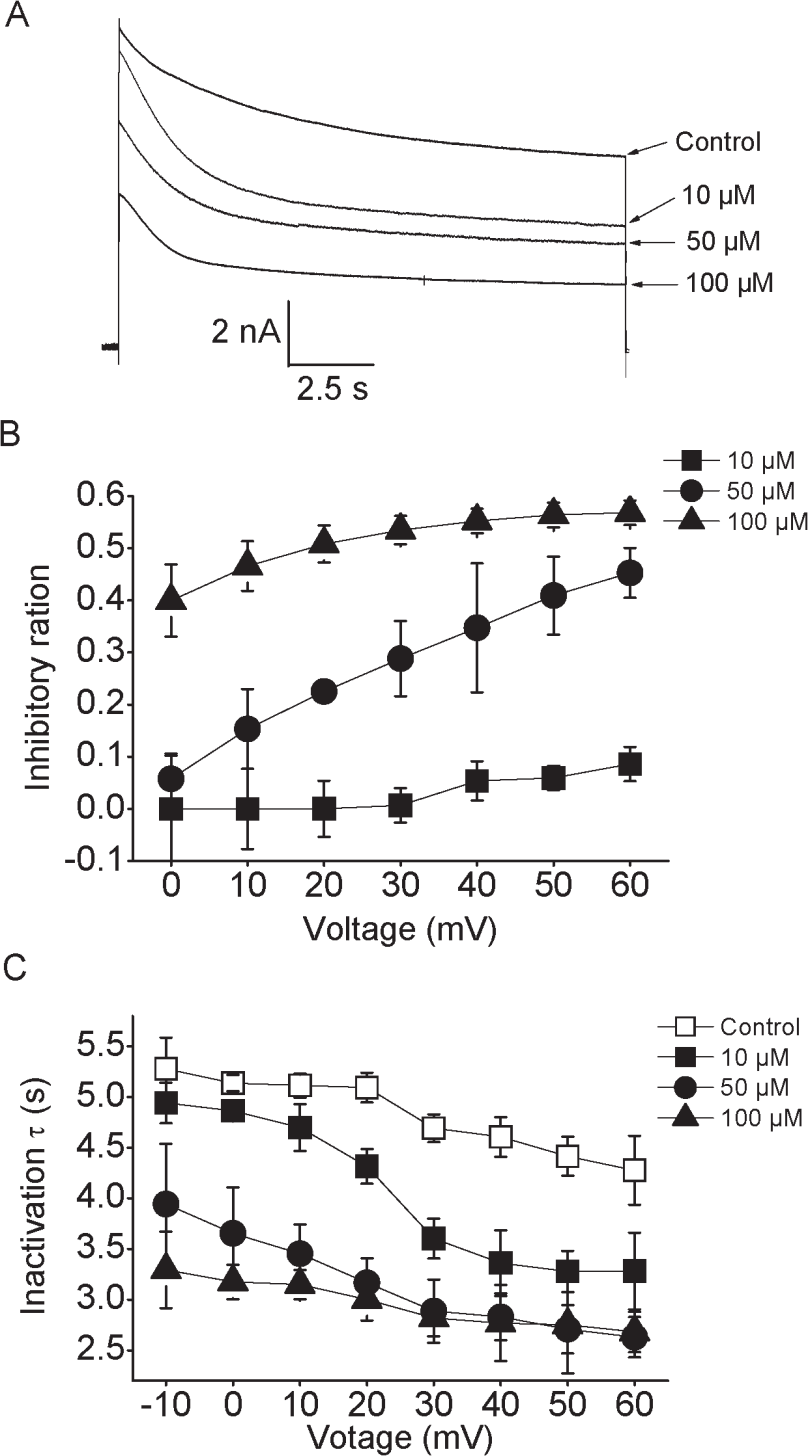
The blockage of Kv2.1 currents by nifedipine in a voltage-dependent manner. (A) The representative current traces were exhibited in the absence and presence of 10, 50 and 100 μM nifedipine. Currents were generated by a 15 s voltage pulse to +60 mV from the holding potential of -80 mV. (B) Different levels of nifedipine inhibited Kv2.1 currents in a voltage-dependent way (n = 8). (C) The voltage-dependent accelerations of inactivation time constants (τ_inact_) were induced by different levels of nifedipine. τ_inact_ was determined by fitting the current elicited by protocol described above using single exponential function (n = 9).

We further examined the voltage-dependent block of nifedipine on the amplitudes and decay time course of Kv2.1 currents. The representative current traces were shown in Fig 4A. The inhibitory percentages of Kv2.1 currents by different concentration of this drug were plotted as a function of test potential (Fig 4B). The increased inhibitions of Kv2.1 currents with depolarization were revealed after applying nifedipine. At +60 mV, 50 μM nifedipine led to a significant decrease in Kv2.1 currents by 40.9 ± 7.5% (n = 8, P<0.05). However, the value of inhibitory percentage at the identical dose of nifedipine became 15.3 ± 7.6% at +10 mV with a statistical difference compared with that at +60 mV (n = 7, P<0.05), suggesting a voltage-dependent blocking.

Previous studies suggested that inactivation time constant (τ_inact_) of Kv2.1 is 3.3–6 s at +40 mV [38, 40, 41]. Therefore, voltage steps with long duration (15 s) to test potentials ranging from -10 to +60 mV were used to elicit the inactivation currents of Kv2.1 channels, and these currents were well fitted with a single exponential function. The values of τ_inact_ in the absence and presence of nifedipine were also plotted as a function of test potential (Fig 4C). Similarly, the inhibitory actions of nifedipine on τ_inact_ of Kv2.1 currents were also dependent on the voltage. Application of 50 and 100 μM nifedipine significantly reduced the τ_inact_ values at the different test potential compared to control (n = 9, P<0.05), suggesting there was an accelerative effect of nifedipine on inactivation of Kv2.1 currents. However, 10 μM nifedipine only significantly accelerated the inactivation of Kv2.1 currents at depolarization potentials (≥ +10 mV). It is well defined that one of the important features of open-channel blocking is the acceleration of the current inactivation [42, 43]. Thus, our data revealed that nifedipine also worked in an open-channel blocking way for Kv2.1 currents.

### Effects of nifedipine on the deactivation of Kv2.1 currents

**Fig 5:**
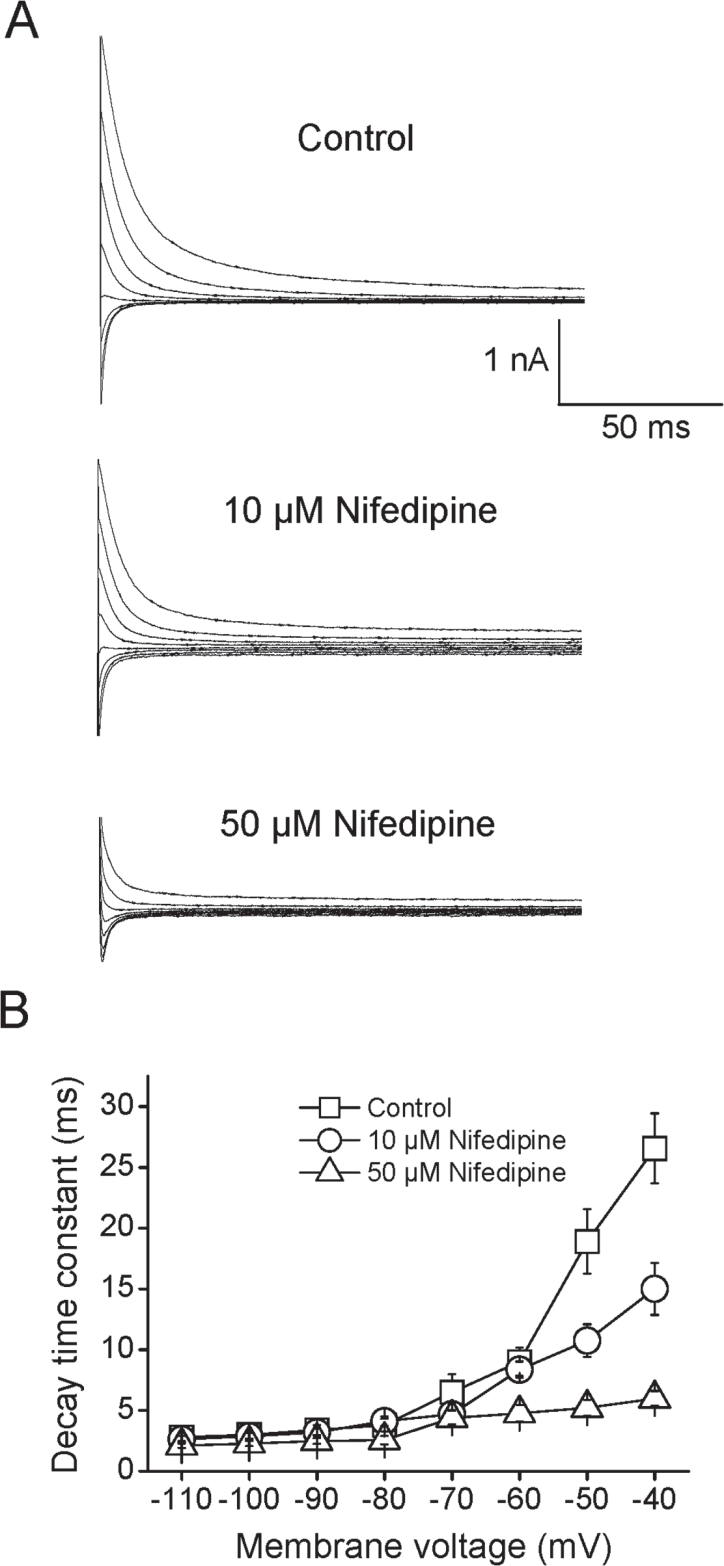
Effects of Nifedipine on the Kv2.1 current deactivation. (A) The representative tail currents of Kv2.1 deactivation were shown for control, 10 μM and 50 μM nifedipine. The tail currents were generated by 500 ms voltage steps to potentials from -110 mV to -40 mV following a conditioning prepulse to +40 mV. (B) The voltage dependence of deactivation time constants of Kv2.1 before and after application of 10 and 50 μM nifedipine. The deactivation time constant was acquired by fitting tail currents using a mono-exponential decay function (n = 9).

Deactivation is a very important state of ion channels during repolarization. The deactivation tail currents of Kv2.1 channels were evoked by voltage steps to test potentials ranging from -110 mV to +40 mV following a conditioning prepulse to +40 mV at a holding potential of -80 mV. Deactivation tail currents in the absence and presence of 10 and 50 μM nifedipine were shown at Fig 5A. Clearly, deactivation time constants (τ_deact_) obtained by exponential fitting were decreased with hyperpolarization (Fig 5B). Treatment with nifedipine significantly reduced the deactivation time courses of tail currents at potentials greater than -60 mV in a concentration-dependent manner (n = 9, P<0.05). It is well known that the open-channel blocker must dissociate from its binding site before channels can close. However, superimposed currents in control and the presence of 10 and 50 μM nifedipine did not exhibit a typical “crossover” phenomenon, which is an indictor for open-channel blocking [23]. These results indicated that the dissociation of nifedipine from deactivating channels was quite rapid.

### Nifedipine altered the inactivation of Kv2.1 currents

**Fig 6:**
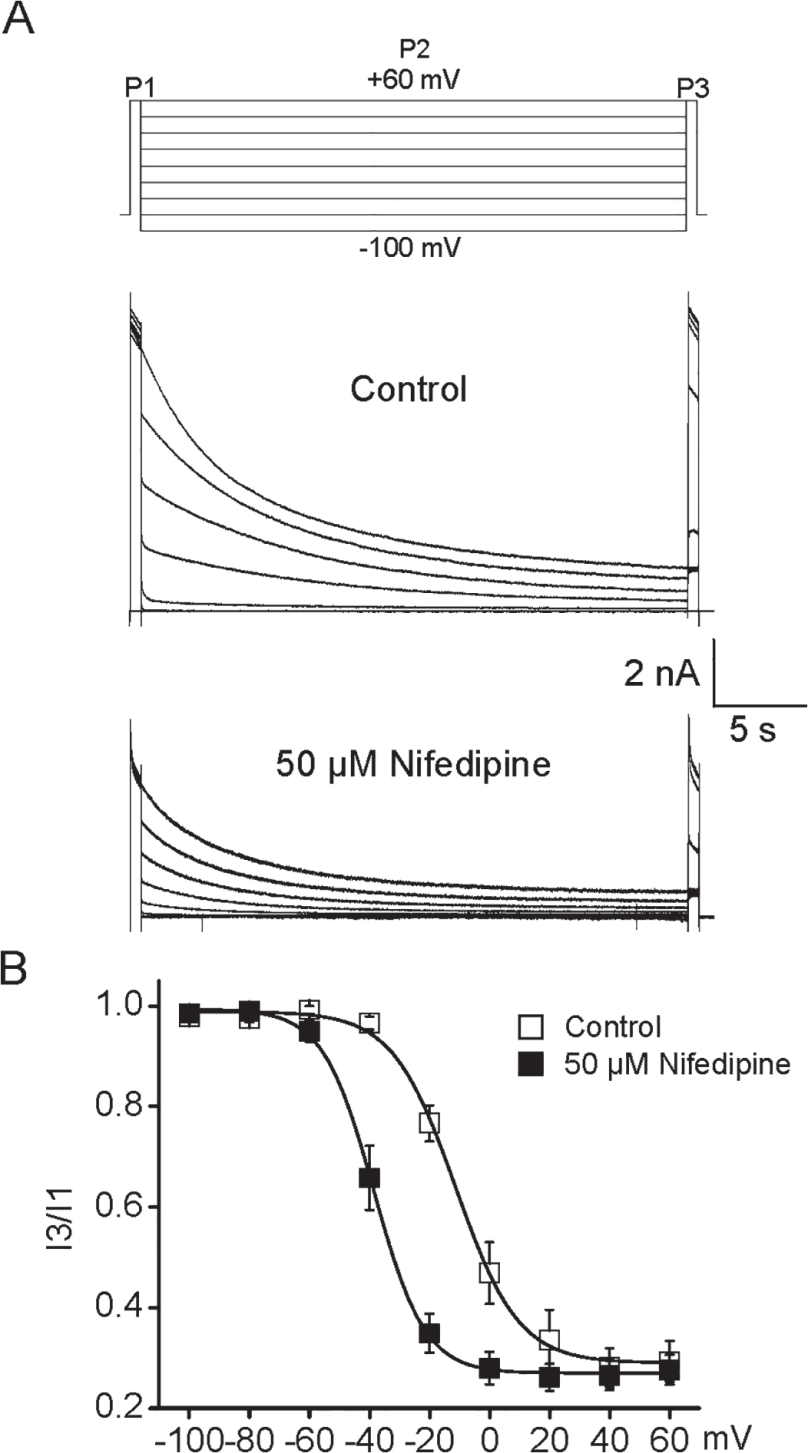
Nifedipine induced a large left shift of the inactivation curve of Kv2.1 currents. (A) The schematic illustrated the three-pulse protocol employed to elicit the currents for building the inactivation curve (top panel). Representative Kv2.1 currents generated by three-pulse protocol in the absence (middle panel) and presence (bottom panel) of nifedipine were shown. (B) The inactivation curves of Kv2.1 currents in the absence and presence of 50 μM nifedipine were constructed by plotting the ration of currents (I3/I1) against the P2 voltage. The steady-state inactivation data were well described by a single Boltzmann with *V*_1/2_ of—11.4 mV and *k* of 8.5 mV in control, as well as *V*_1/2_ of—38.5 mV and *k* of 10.1 mV in the presence of 50 μM nifedipine (n = 8).

We next examined the action of nifedipine on slow inactivation of Kv2.1 currents. To avoid cumulative inactivation, a three-pulse (3P) protocol was used for eliciting the inactivation currents [44]. At the beginning of each record, currents elicited by an additional prepulse (P1) to +60 mV were taken as a control for accumulation of inactivation. A 15 s long voltage pulses (P2) to potentials between -100 and +60 mV were conducted to induce inactivation. Currents acquired during the final pulse (P3) to test potential of +60 mV were normalized to those from P1. Inactivation was measured as the ratio of the currents at +60 mV (I3/I1). Previous studies indicated that the voltage dependence of steady-state inactivation relationships for Kv2.1 channels showed a clear U-shape in some studies [40, 44]; however, it is absent in our study (Fig 6A) and other studies [45, 46].

As shown in Fig 6B, there was little inactivation of Kv2.1 currents when the conditioning prepulses were more negative than -60 mV; however, inactivation actually was accelerated with applied depolarization pulse. Kv2.1 currents were almost completely inactivated by conditioning prepulses positive to +40 mV. The steady-state inactivation data for outward currents in control were well described by a single Boltzmann with *V*_1/2_ value of -11.4 ± 0.9 mV and *k* value of 8.5 mV. In the presence of 50 μM nifedipine, the inactivation curve was significantly shifted to more negative potentials with *V*_1/2_ value of -38.5 ± 0.4 mV and *k* value of 10.1 mV (n = 8, P<0.05). Nifedipine led to a 27 mV hyperpolarizing shift of the inactivation curve without significant effect on the slope factors. It is rather interesting since nifedipine barely changed the inactivation curves of currents mediated by cloned and native Kv channels in previous studies [24, 27, 47].

### Nifedipine appears not to be a direct channel pore blocker

**Fig 7:**
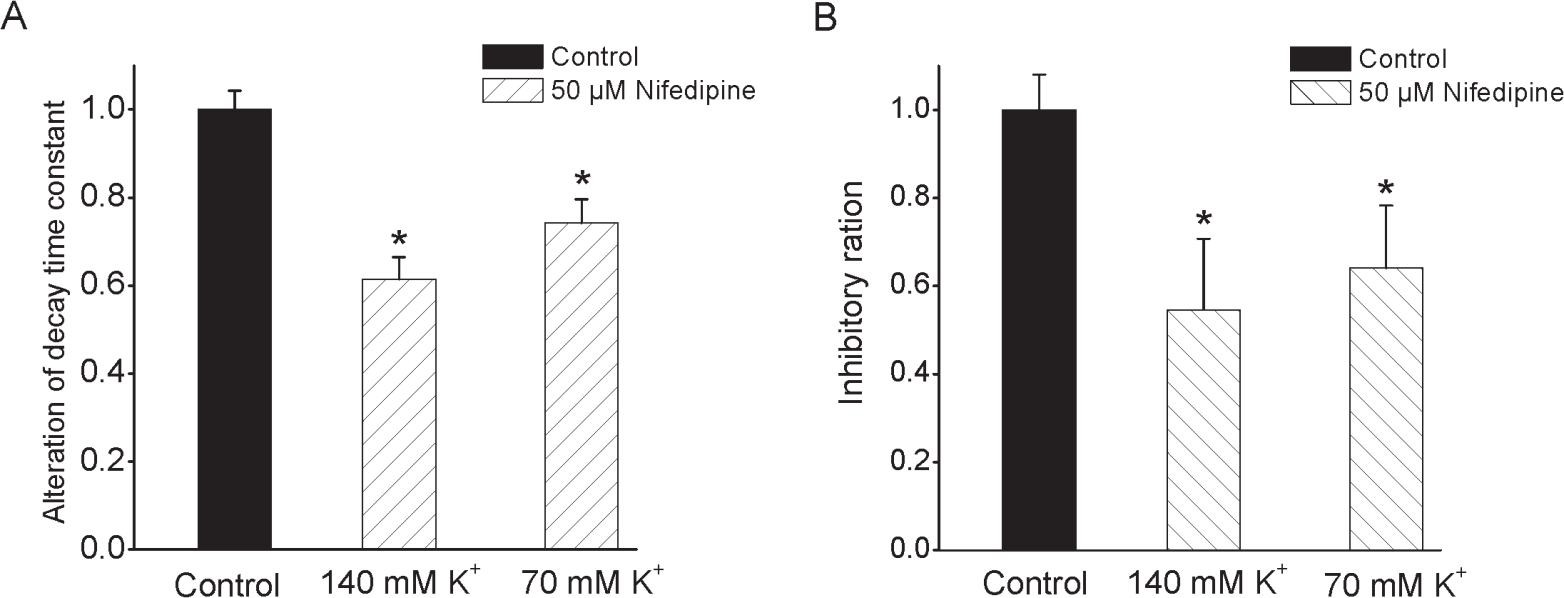
Nifedipine block was unaffected by intracellular K^+^ concentration. (A) Exposure to 50 μM nifedipine significantly accelerated the inactivation time course of Kv2.1 currents in the presence of 140 and 70 mM pipette K^+^. However, there was not significant difference for inactivation time course between 140 and 70 mM pipette K^+^ (n = 9). (B) The percentage block of Kv2.1 currents by 50 μM nifedipine in the presence of 70 and 140 mM intracellular K^+^ (n = 9).

To demonstrate whether nifedipine blocked Kv2.1 channels by directly occluding the channel pore, we tested the effect of intracellular K^+^ concentration on nifedipine actions. The hypothesis is that nifedipine and K^+^ must encounter each other in the pore itself if this drug directly occludes the pore. Therefore, a decreased intracellular level of K^+^ could cause an increased effect of nifedipine on Kv2.1 channels. As shown in Fig 7 A and 7 B, the intracellular K^+^ concentration of 70 mM did not significantly alter the inhibitory percentage and inactivation time course of nifedipine compared to those under the condition of intracellular 140 mM K^+^, suggesting that nifidipine was not likely a direct pore obstructer (n = 9, P>0.05).

### Nifedipine acted on recovery of Kv2.1 from inactivation

**Fig 8:**
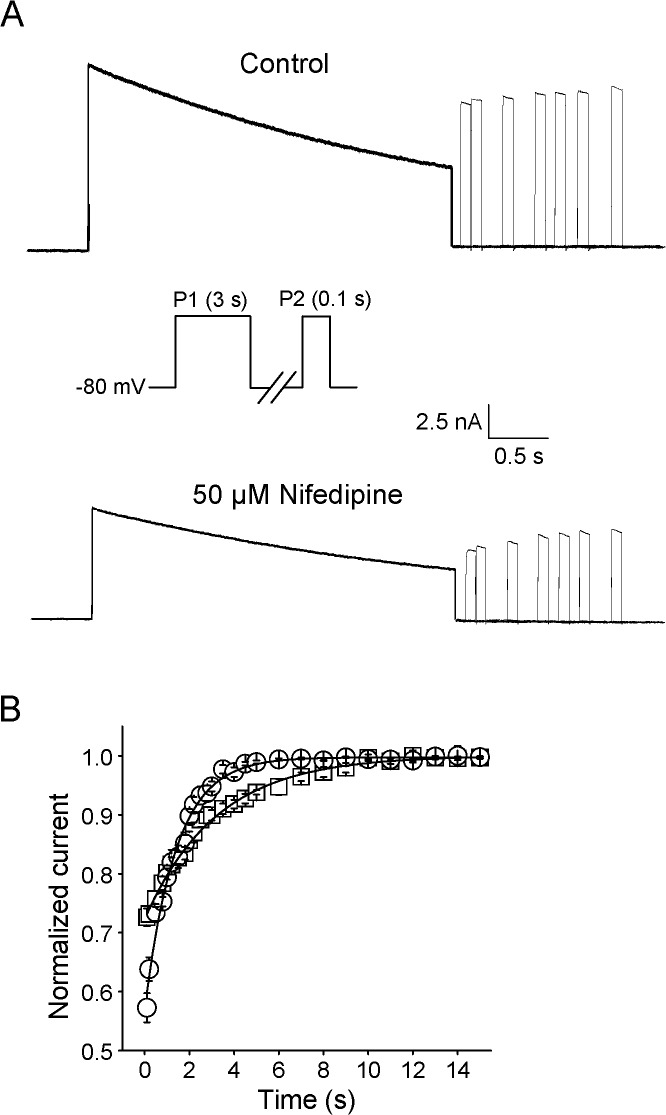
Nifedipine shorten the recovery of Kv2.1 currents. (A) Typical recovery waveforms of Kv2.1 currents were recorded in the absence (upper panel) and presence of 50 μM nifedipine (bottom panel). Currents were elicited by a 100 ms test potential to +40 mV following a 3 s conditioning prepulse to +40 mV with varying recovery intervals from 100 ms to 15 s. The experimental protocol is illustrated between the records. (B) Normalized recovery currents were plotted for control and the presence of 50 μM nifedipine. Data were fitted by a mono-exponential function to obtain the recovery time constant (n = 7).

Further measurements were conducted to explore the effects of nifedipine on the recovery rate of Kv2.1 channels. Currents were first evoked by a 3 s long pulse to +40 mV for activation and inactivation. Subsequently, Kv2.1 channels were hyperpolarized to -80 mV for varying time ranging from 100 ms to 15 s, and finally stepped back to +40 mV to assess the extent of recovery. Data were well fitted by a mono-exponential function to build the recovery curve shown in Fig 8. The recovery time constants (τ_recor_) in the absence and presence of 50 μM nifedipine were 3.4 ± 0.3 s and 1.4 ± 0.1 s, respectively, suggesting a acceleration of 58.8% in the recovery rate of Kv2.1 channels by nifedipine (n = 7, P<0.05). Previous results revealed that the dissociation of an open-channel blocker could delay recovery from inactivation [48]. Nevertheless, the opposite effects of nifedipine presented here suggested that open-channel binding of nifedipine was inappropriate interpretation for its effect on recovery of Kv2.1 channels. Alternatively, nifedipine appeared to accelerate the recovery of Kv2.1 channels by regulating channel gating.

## Discussion

Previous studies reported that the dihydropyridine Ca^2+^ channel blocker nifedipine inhibited a variety of K^+^ channels, including Kv1.1, Kv1.2, Kv1.3, Kv1.5, Kv3.1, and Kv4.3 channel, heterologously expressed in mammalian cell lines and/or Xenopus oocytes [24, 49]. So far, there were not any reports about the effects of this drug on Kv2.1 channels, which mediated delayed rectifier K^+^ currents with slow inactivation. In the study here, we demonstrated that nifedipine substantially blocked the Kv2.1 channels expressed in HEK293 cells in concentration- and voltage-dependent manners.

The finding of an acceleration of current inactivation suggested an open-channel binding of nifedipine to Kv2.1 channels. Similarly, nifedipine also exerted an inhibitory effect on Kv4.3 and Kv1.5 by binding those channels at the open state [23, 24]. Besides accelerating the current inactivation [42, 43], the open-channel blocker also produced a “crossover” of deactivation tail current due to the prolongation of time course of deactivation [48]. However, crossover of tail currents was absent in the presence of different dose of nifedipine. Experimental evidence in our study revealed that nifedipine also inhibited Kv2.1 currents in a closed-channel blocking way. For instance, the treatment with nifedipine delayed the activation time course of Kv2.1 currents, coinciding with the slowed activation by the closed-channel blocker [39].

In many reports, Kv2.1 channels exhibit a U-shaped voltage-dependence of inactivation, which was considered as preferential inactivation from preopen closed states [40, 44, 50]. However, this phenomenon was not clearly observed in other studies [45, 46]. Although voltage clamp protocol for measurement of inactivation used in our study was similar as that used by Cheng et al [50], we did not record the typical U-shaped of inactivation after repeated testing. The exact reason is unknown and it may be attributed to the difference between us such as the expression system and solutions.

Nifedipine induced a substantial leftward shift of steady-state inactivation of Kv2.1 currents. Previous works suggested that nifedipine did not change the inactivation curves of cloned Kv4.3 channels [24]. In addition, the steady-state inactivations of both transient outward K^+^ currents (I_to_) in human atrial myocytes [27] and unidentified Kv currents in rat DRG neurons [47] were also barely affected by nifedipine. It appears likely that Kv2.1 channels are the first member of Kv channel superfamily, of which steady-state inactivation was altered by nifedipine. The more interesting thing is that the potency (27 mV) of hyperpolarizing shift of the half-maximal inactivation potential by nifedipine is relatively large. However, the mechanism(s) underlying this phenomenon is unknown.

Nifedipine is expected to in its neutral form at a physiological pH of 7.4 due to a low pKa ≤ 1.0 [51]. As an uncharged drug, it seems unlikely that nifedipine blocked the Kv2.1 channels in a voltage-dependent manner. Nevertheless, previous work by Jacobs et al [52] suggested that the binding site of uncharged blockers such as nifedipine may be coupled to voltage-dependent process, and finally voltage-dependent blocking was conferred to those uncharged drugs.

It has been reported that nifedipine blocked I_to_ and IKur in human atrial myocytes with IC_50_ values of 26.8 and 8.2 μM, respectively [27]. For hKv1.5 current expressed in HEK cells and mouse fibroblasts, the values of IC_50_ are 6.2 μM [23] and 92 μM [49], respectively. In the present study, the value of IC_50_ is 37.5 μM for nifedipine-induced inhibition of Kv2.1 channels, and it is only lower than that for nifedipine-induced inhibition of hKv1.5 expressed in mouse fibroblasts. Furthermore, the value of IC_50_ in this study is higher than the block concentration (0.05–0.2 μM) for L-type Ca^2+^ currents in guinea-pig myocardium [53]. Although it needs relative high levels of nifedipine to block Kv2.1 channels expressed in HEK293 cells, the blocking effects of this chemical on Kv2.1-mediated currents may be different in distinct cells. It was worth noting that nifedipine produced a 27 mV hyperpolarizing shift of the inactivation curve. This potent shift is expected to cause a strong inactivation in outward delayed rectifier K^+^ currents mediated by Kv2.1 channels at repolarization phases. Subsequently, Action potential durations of a variety of cells such as cardiomyocytes and neuron were prolonged, and the alteration of pathophysiological functions of these cells could occur.

In summary, the present study has provided the first evidence that the Ca^2+^ antagonist nifedipine block the Kv2.1 channels expressed in HEK293 cells. The huge left shift of the inactivation curve induced by nifedipine may result in more inactivation for Kv2.1 currents in depolarization potentials. Our data will be helpful to understand the diverse effects exerted by nifedipine on various Kv channels.
